# The Author-Level Metrics Study: An Analysis of the Traditional and Alternative Metrics of Scholarly Impact for Neurosurgical Authors

**DOI:** 10.7759/cureus.27111

**Published:** 2022-07-21

**Authors:** Sukumar Kalvapudi, Subeikshanan Venkatesan, Rishab Belavadi, Varun Anand, Venkatesh S Madhugiri

**Affiliations:** 1 Neurosurgery, Jawaharlal Institute of Postgraduate Medical Education and Research (JIPMER), Pondicherry, IND; 2 Surgery, Jawaharlal Institute of Postgraduate Medical Education and Research (JIPMER), Pondicherry, IND; 3 Neurosurgery, National Institute of Mental Health and Neuro Sciences (NIMHANS), Bangalore, IND

**Keywords:** citations, impact matrix, neurosurgery, alternative metrics, h-index, scopus, google scholar, researchgate

## Abstract

Background and objective

There is a paucity of information regarding the concordance of traditional metrics across publicly searchable databases and about the correlation between alternative and traditional metrics for neurosurgical authors. In this study, we aimed to assess the congruence between traditional metrics reported across Google Scholar (GS), Scopus (Sc), and ResearchGate (RG). We also aimed to establish the mathematical correlation between traditional metrics and alternative metrics provided by ResearchGate.

Methods

Author names listed on papers published in the Journal of Neurosurgery (JNS) in 2019 were collated. Traditional metrics [number of publications (NP), number of citations (NC), and author H-indices (AHi)] and alternative metrics (RG score, Research Interest score, etc. from RG and the GS i10-index) were also collected from publicly searchable author profiles. The concordance between the traditional metrics across the three databases was assessed using the intraclass correlation coefficient and Bland-Altman (BA) plots. The mathematical relation between the traditional and alternative metrics was analyzed.

Results

The AHi showed excellent agreement across the three databases studied. The level of agreement for NP and NC was good at lower median counts. At higher median counts, we found an increase in disagreement, especially for NP. The RG score, number of followers on RG, and Research Interest score independently predicted NC and AHi with a reasonable degree of accuracy.

Conclusions

A composite author-level matrix with AHi, RG score, Research Interest score, and the number of RG followers could be used to generate an "Impact Matrix" to describe the scholarly and real-world impact of a clinician’s work.

## Introduction

The endeavor to mathematically quantify the impact of individual clinicians and researchers on their respective fields has led to the creation of several author- and institute-level metrics. The "traditional" author-level metrics include the number of papers published by an author [number of publications (NP)], the number of citations (NC) accrued by an author, and metrics that combine the preceding two, such as the author H-index (AHi), G-index, and i-10 index. Several academic databases such as Web of Science, Google Scholar (GS), Scopus (Sc), Publons, and ResearchGate (RG) provide the traditional metrics at the individual author level, and they have become widely used. However, the major limitation of the entire class of citation-based metrics has always been their inability to capture the real-world impact of a body of research, which may not necessarily be reflected by the NC received by a paper or an author [1,2,3]. This is especially true for studies with major design flaws that still manage to get published and cited.

Knowledge-sharing paradigms have evolved and, currently, the lion's share of knowledge-sharing occurs on "non-traditional" sites on the internet, examples being social media platforms and preprint servers. As a response to this shift, several bibliometric databases and social networking websites have developed "alternative" metrics. The alternative metrics incorporate elements that could possibly capture the real-world impact of research more effectively than citation-based metrics. For instance, the scholarly social network ResearchGate provides an array of novel metrics such as the RG score, Research Interest score, and RG reads. However, the major criticism leveled at the alternative metrics and the sites that provide them has been the lack of transparency regarding the algorithms used to calculate the metrics, thus precluding reproducibility [4,5,6]. Populism in science is a concerning issue and, therefore, there is a need to scientifically investigate these upcoming alternative metrics.

Traditional and alternative metrics are increasingly being used to inform performance evaluations, promotions, funding, and the grant of tenure to faculty members in academic institutions. However, these metrics may not be entirely suited to the purposes they are being used for. For instance, the values of both traditional and alternative metrics lack congruence across several databases. Moreover, the algorithms used to compute the alternative metrics are considered a black box. Therefore, it becomes important to evaluate the accuracy and congruence of the traditional metrics reported across databases and to establish how the alternative metrics correlate with the conventional metrics. Significant variations in these metrics across databases or improper use of these metrics to inform decisions could adversely impact career growth, especially for early-career clinicians and researchers. Standardization of metrics is essential for making comparisons across databases.

Neurosurgical research is a niche field, with a relatively limited pool of authors and readers. There is a paucity of data regarding the validity of the alternative metrics for neurosurgeons vis-à-vis the traditional metrics [7,8]. In this study, we compared the traditional metrics reported across different platforms for a cohort of neurosurgery authors who had published their work in the principal neurosurgical journal, the Journal of Neurosurgery (JNS). In addition, we also analyzed the mathematical correlations of the alternative metrics reported by RG and GS with the traditional author-level metrics for neurosurgery.

## Materials and methods

Data collection

The list of authors who were listed on papers published in JNS in the year 2019 was obtained by querying the PubMed database, using the timeline and journal filters. The RG, GS, and Sc profiles of the listed authors (where available) were accessed and the following data points were collated from each site.

Traditional Metrics

These entailed NP, NC, and AHi. We also obtained the i10-index for the authors from GS.

Alternative Metrics

From RG: RG score, Research Interest, RG reads, follower count, following count, number of recommendations, number of questions, and number of answers. We also obtained data on author demographics: authors' country of origin and affiliation (university and department) as listed on RG.

We used metrics from RG since they are freely available to all registered users. Other novel metrics, such as the Altmetric score, for instance, are not freely available. Authors who did not have an RG profile were excluded from the study.

Statistical analysis 

This study incorporated a two-part analysis. In the first part, we analyzed the congruence between the traditional metrics reported across three academic sites: RG, GS, and Sc. Agreement between the traditional metrics reported on the three databases was estimated using the intraclass correlation coefficient (single-rater, absolute-agreement, two-way mixed effects model) and Bland-Altman (BA) plots. Paired sample median differences were analyzed using the Wilcoxon rank-sum test. Differences between medians of multiple groups were evaluated using the Kruskal-Wallis test.

The second set of analyses focused on the alternative metrics. Firstly, bivariate correlations were used to analyze the degree of association between the traditional metrics and alternative metrics. Next, the alternative metrics that correlated with the traditional metrics reported in the Sc database in the preceding set of analyses were entered into a stepwise backward elimination multivariate regression model, to identify the alternative metrics that could independently predict the traditional metrics. A separate regression analysis was carried out with each Sc traditional metric set as the dependent variable. The alpha value was set a priori at 0.05 for statistical significance. All analyses were carried out using SPSS Statistics v26 (IBM, Armonk, NY).

## Results

One thousand eight hundred and two of 5589 authors (32%) who were listed on papers published in JNS in 2019 were included in the final analysis, in accordance with the prespecified inclusion criteria. Based on authors who had profiles on various databases, the number of entries analyzed from each database was as follows: Sc: 1772, RG: 1802, and GS: 1172. Thus, a total of 4751 author profiles were analyzed across the analyzed databases.

The traditional metrics

Descriptive data pertaining to the traditional metrics are listed in Table 1. Traditional metrics obtained from Sc were used as the benchmark for comparing the corresponding metrics from GS and RG.

**Table 1:** Analysis of the traditional metrics

	Number of publications			Number of citations			Author H-index		
	Google Scholar	Scopus	ResearchGate	Google Scholar	Scopus	ResearchGate	Google Scholar	Scopus	ResearchGate
Number of author profiles	1176	1773	1802	1176	1772	1799	1176	1773	1796
Median	70.5	47	65	837.5	585	673	14	12	12
Interquartile range	127	90	122	2665.5	2084.25	2308	20	18	18
Minimum	1	1	1	1	0	1	1	0	1
Maximum	2927	1245	1534	103,276	130,476	76,799	150	139	127

**Figure 1:**
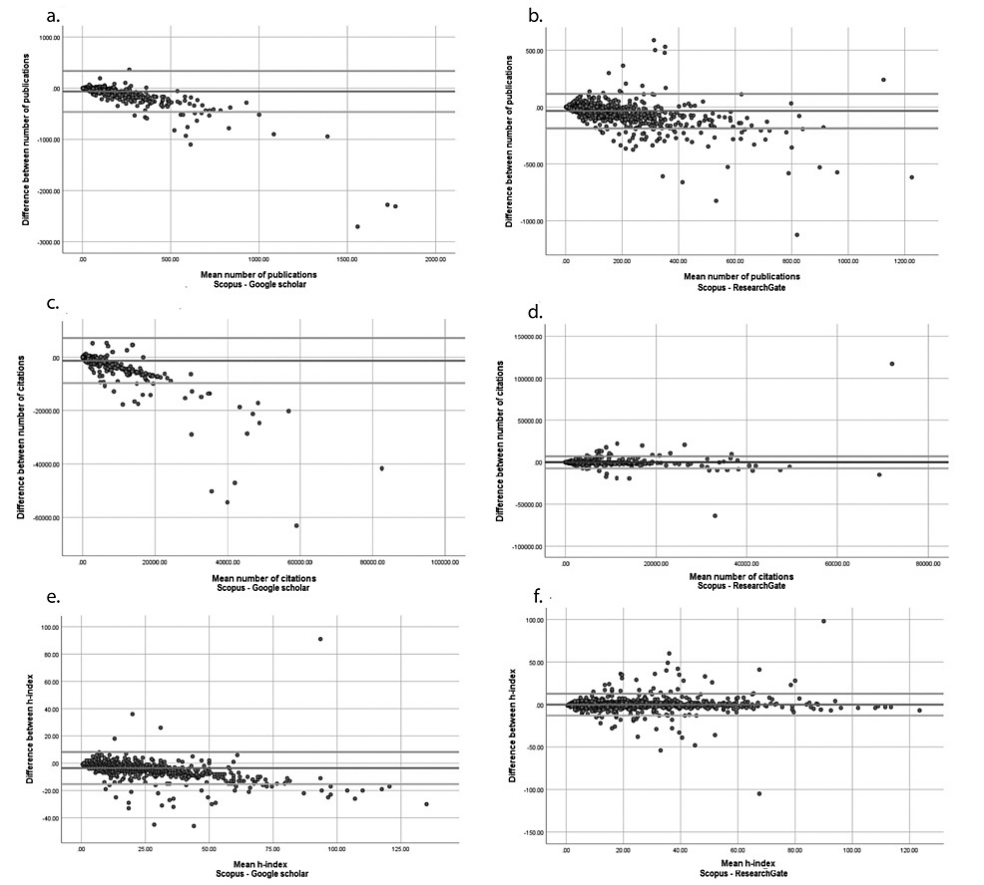
Bland-Altman (means vs. differences) plots of traditional metrics from Scopus vs. the same metrics from Google Scholar and ResearchGate The middle line represents the mean difference. The top and bottom lines represent the +2SD and -2SD of this difference respectively. The agreement between the number of publications as reported on Scopus and Google Scholar (a) and Scopus and ResearchGate (b) was good at lower publication counts. The agreement between the number of citations received by an author as reported on Scopus and Google Scholar (c) and Scopus and ResearchGate (d) was good for the latter pair but not for the former. The agreement between the author H-index as reported on Scopus and Google Scholar (e) and Scopus and ResearchGate (f) was excellent

Agreement between NP as listed on Sc and GS was found to be moderate, with an ICC of 0.562 (p<0.0001, 95% CI: 0.449-0.648). The NP values from RG agreed well with the Sc-NP values, with an ICC of 0.809 (p<0.0001, 95% CI: 0.722-0.838). The BA plots for Sc-NP vs. GS-NP and Sc-NP vs. GS-NP showed an increased degree of scatter (outside the ±1.96 SD lines) as NP increased. The concordance between the databases was better at lower values of NP (Figures 1a, 1b).

There was good agreement of the NC counts across databases. The ICC value for the agreement of NC-Sc with NC-GS was 0.784 (p<0.0001, 95% CI: 0.732-0.824), while the ICC for the agreement of NC-Sc with NC-RG was 0.800 (p<0.0001, 95% CI: 0.782-0.816). The BA plots are displayed in Figure 1c and Figure 1d.

The agreement between AHi values was excellent across databases. The ICC for agreement of AHi-Sc with AHi-GS was 0.926 (p<0.0001, 95% CI: 0.836-0.959). The ICC for agreement of AHi-Sc with AHi-RG was 0.928 (p<0.0001, 95% CI: 0.921-0.934). The BA plots for AHi are displayed in Figure 1e and Figure 1f.

GS provides author-level i10-index values. The median i10-index for this cohort of JNS authors was 18 (n=1120, IQR=49). The paired sample median AHi from GS was significantly lower than the i10-index (p<0.0001). There was a strong positive correlation between the GS i10-index and Sc-AHi (Spearman’s ⍴=0.938). As expected, the GS i10-index also correlated strongly with the Sc-NP (Spearman’s ⍴=0.899) and Sc-NC (Spearman’s ⍴=0.925).

The alternative RG metrics

**Figure 2:**
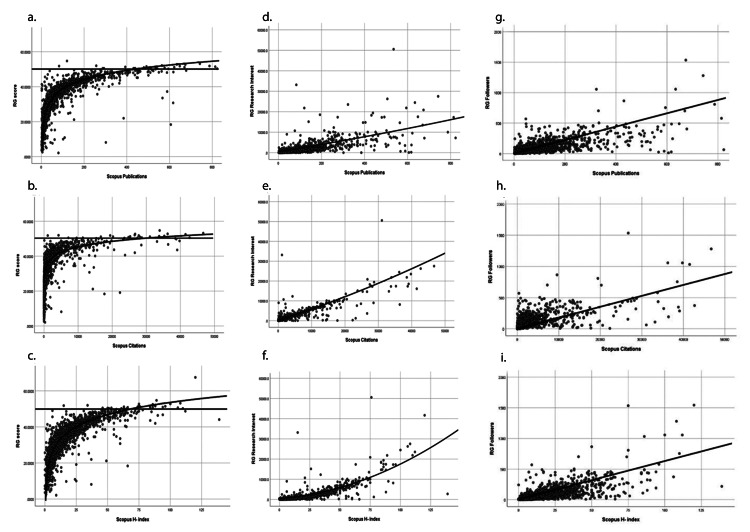
The mathematical relationship between the traditional and alternative metrics The RG score displayed a logarithmic relationship with the number of publications (a), the number of citations received by an author (b), and the author H-index (c). There was a good clustering of data points around the regression line that depicts the relation between the RG Research Interest score and the number of publications (d), the number of citations received by an author (e), and the author H-index (f) as reported on Scopus. A similarly strong correlation was seen between the number of followers on RG and the number of publications (g), the number of citations received by an author (h), and the author H-index (i) as reported on Scopus

Descriptive data pertaining to the RG metrics are listed in Table 2. The RG score correlated well with the traditional metrics from Sc: NP (Spearman’s ⍴=0.91, p<0.001), NC (Spearman’s ⍴=0.86, p<0.001), and AHi (Spearman’s ⍴=0.88, p<0.001) (Table 3). The RG score displayed a logarithmic relation with NP-Sc, NC-Sc, and AHi-Sc, with the RG score approaching an asymptote at RG score >50 (Figures 2a, 2b, 2c).

**Table 2:** Analysis of the ResearchGate metrics

	RG score	Research Interest	RG reads	Questions	Answers	Following	Followers
Number of author profiles	1798	1802	1802	1802	1802	1799	1801
Median	32.12	422.45	4692.5	0	0	29	48
Interquartile range	15.12	1270.83	9267	0	0	45	86
Minimum	2.11	0.2	10	0	0	0	0
Maximum	67.48	50,538	262,752	69	28	1048	1544

**Table 3:** Correlation matrix of the RG alternative metrics with the traditional metrics from Scopus

ResearchGate alternative metric	Scopus traditional metric	Coefficient of correlation (Spearman’s rho)	P-value
RG score	Number of publications	0.901	<0.001
	Number of citations	0.861	<0.001
	Author H-index	0.878	<0.001
RG Research Interest	Number of publications	0.853	<0.001
	Number of citations	0.931	<0.001
	Author H-index	0.916	<0.001
RG reads	Number of publications	0.813	<0.001
	Number of citations	0.783	<0.001
	Author H-index	0.795	<0.001
RG questions	Number of publications	-0.022	0.349
	Number of citations	-0.032	0.172
	Author H-index	-0.039	0.101
RG answers	Number of publications	0.036	0.127
	Number of citations	0.016	0.489
	Author H-index	0.017	0.472
RG followers	Number of publications	0.758	<0.001
	Number of citations	0.744	<0.001
	Author H-index	0.749	<0.001
RG following	Number of publications	0.247	<0.001
	Number of citations	0.194	<0.001
	Author H-index	0.203	<0.001

We then analyzed the correlation between the individual components that were utilized to compute the RG Score (as described on the RG site) and the RG score itself. The listed components of the RG score were as follows: NP, the number of RG followers, the number of questions asked, and the number of answers provided on the RG site. The number of RG followers showed a strong positive correlation with the RG score (Spearman’s ⍴=0.817). However, neither the number of questions asked (Spearman’s ⍴=0.002) nor the number of questions answered (Spearman’s ⍴=0.059) correlated well with the RG score.

"Research Interest" has been defined as a metric that assesses the interest of scientific peers in an author’s research. Research Interest correlated strongly with NP (⍴=0.85, p<0.001), NC (⍴=0.93, p<0.001), and AHi (⍴=0.91, p<0.001) from Sc (Table 3). The correlation between Research Interest and Sc-NP, NC, and AHi is displayed in Figures 2d-2f. The component metrics of the Research Interest score were listed as RG reads, recommendations, and NC-RG. Research Interest had a strong positive linear correlation with NC-RG (⍴=0.993) and RG reads (⍴=0.877) and a weaker correlation with recommendations (⍴=0.61).

RG reads displayed strong correlations with Sc-NP, Sc-NC, and Sc-AHi (Table 3). Similarly, the number of RG followers and the number following an author both correlated with the three traditional Sc metrics (Table 3). Figures 2g-2i display the correlation between RG followers and Scopus NP, NC, and AHi. However, the number of questions asked and the number of answers provided did not correlate with the traditional metrics (Table 3).

From the new to the old: deriving the traditional metrics

The traditional metrics from Sc were considered to be the dependent variables for this set of regression analyses (vide supra). A regression equation with adequate statistical significance could be generated (p<0.0001, R2=0.752) to predict the Sc-AHi:

Sc-AHi = -9.501 + [0.712 × (RG score)] + [0.002 × (Research Interest)] + [0.016 × (followers)]

Similarly, the equation to predict the Sc-NC (p<0.0001, R2=0.607) was as follows:

Sc-NC = -854.5 + [35.49 × (RG score)] + [1.29 × (Research Interest)] + [4.29 × (followers)]

Thus, the RG score, the number of followers on RG, and the Research Interest score appeared to predict both NC and AHi with a reasonable degree of accuracy.

Global geographic analysis

Author affiliations were broadly grouped according to the continents. The contribution of each continent to the 2019 author pool of JNS was as follows: Africa: 0.5%, Asia: 19.8%, Australia-Oceania: 0.8%, Europe: 28.2%, North America: 48.6%, and South America: 2.1%. Authors from Australia-Oceania had the highest median GS-i10 (35) whereas Asian authors had the highest median Sc-NP (52). European authors had the highest NC and AHi on Sc and also had the highest median composite RG metrics (RG score and RG Research Interest). South American authors had the highest median scores for those RG metrics that were wholly dependent on social/peer engagement (RG recommendations, RG reads, RG followers, and RG following). With the exception of the i10-index, Sc-NP, and RG score, the other traditional and alternative metrics were statistically different between the continents (Table 4).

**Table 4:** Continent-wise comparison of the traditional and alternative metrics The last column displays the results of the Kruskal-Wallis test to evaluate the differences between the median values for each continent. The values in bold font in each row denote the highest median value for that category. The categories with significant differences between continents have been highlighted in bold font (last column) GS: Google Scholar; Sc: Scopus, NP: number of publications; NC: number of citations; AHi: author H-index, RG: ResearchGate; IQR: interquartile range

Category	Metric (median, IQR)	Africa (n=9)	Asia (n=356)	Australia-Oceania (n=15)	Europe (n=508)	North America (n=876)	South America (n=38)	χ^2^ (p-value)
Traditional	GS-i10	9 (10)	20.5 (47)	35 (58)	21 (44)	17 (49)	13 (61)	3.5 (0.62)
Traditional	Sc-NP	18.5 (26)	52 (77)	40 (95)	51 (89)	45 (95)	34.5 (106.5)	7.4 (0.19)
Traditional	Sc-NC	123.5 (156.5)	466 (1398)	455 (1763)	751 (2164)	586 (2726.5)	224 (980.5)	22.4 (0.0004)
Traditional	Sc-AHi	6 (4.5)	11 (15)	12 (18)	14 (18)	12 (20)	8 (14)	18.1 (0.003)
Alternative	RG score	30.27 (5.91)	32 (13.14)	28.07 (23.44)	32.8 (14.65)	32.22 (16.7)	29.93 (20.74)	9.44 (0.09)
Alternative	RG Research Interest	79.1 (115.5)	332.8 (868.8)	391.3 (1326.5)	500.75 (1391.8)	420.7 (1514.6)	244.8 (917.7)	20.7 (0.0009)
Alternative	RG recommendations	7 (5)	8 (15)	3 (55)	20 (42)	10 (22)	35 (75)	103.2 (0.0001)
Alternative	RG reads	1922 (1404)	4113.5 (7398)	5425 (8620)	5120.5 (10,419)	4446.5 (9315)	5842 (22,235)	20.3 (0.001)
Alternative	RG following	40 (46)	26 (38)	40 (67)	38 (53)	26 (44)	48.5 (73)	34.9 (0.0001)
Alternative	RG followers	18 (17)	36.5 (64.5)	47 (135)	59 (94.5)	47 (91)	69.5 (124)	40.2 (0.0001)

## Discussion

Traditional and novel metrics are increasingly being used to inform decisions regarding recruitment, career advancement, grants, etc. While the shortcomings of the traditional citation-based metrics are well known, they have the advantage of being objective and easily reproducible. The novel social media-based metrics are unknown unknowns; they are neither easily computed nor has their exact correlation with the traditional metrics been established hitherto. In the present analysis, we were able to demonstrate clear correlations between the novel and traditional metrics. These correlations add credibility to the use of novel metrics in the context of performance and impact evaluation.

Traditional metrics from Sc were considered to be the benchmarks for all comparisons across this analysis. Both Sc and Clarivate’s Web of Science are widely considered to be the standard sources of scholarly metrics since these databases are curated by independent subject experts and only include information from reliable sources of scholarly repute [9,10,11].

The traditional metrics

We found that the values of the traditional metrics reported on Sc were consistently lower than in the other two databases analyzed (GS and RG), possibly owing to the careful curation of data sources. GS consistently reported the highest values for the analyzed traditional metrics; this finding has been reported hitherto in several studies (Table 1) [2,12].

The agreement between GS and Sc (ICC=0.597) and RG and Sc (ICC=0.809) was reasonably good for NP, especially at lower NP values. However, for more prolific authors (higher NP), the agreement between Sc and the other two databases broke down (Figures 1a, 1b). A similar trend was seen in the BA plots for agreement of the NC between GS and Sc (ICC=0.784) - at higher citation counts (higher NC), the agreement was poorer (Figure 1c). The NC-RG, however, showed good agreement with the NC-Sc (ICC=0.800) and the BA plot showed that most measured data points were between the ±1.96 SD lines (Figure 1d).

Thus, neither GS nor RG displayed good agreement with Sc for more prolific authors (high NP) whereas GS had a poor agreement with Sc for the more impactful (higher cited) authors. On GS, this could be due to the fact that the GS database is populated by a scientific search-engine algorithm that automatically includes data from all journals (indexed, open access, and popular science), conference proceedings, books, theses, reports, local press, electronic sources, etc. Several of these sources are not verified independently by experts and could cause errors in the NP. On the other hand, lack of expert curation renders the platform susceptible to manipulation of citation counts, counting spam articles in its listings; thus, the citation metrics could potentially be inflated [13,14,15]. RG, on the other hand, is not transparent about how data pertaining to author-level metrics are collected. Information provided on the site states that citation data is imported from various (unspecified) sources. Moreover, users are allowed to directly upload research items and publications (including preprints) on RG and these are possibly counted towards NP and NC calculations. Thus, both RG and GS may have lower accuracy for NP and NC values due to systematic issues. 

There was excellent agreement between GS and Sc (ICC=0.926) and RG and Sc (ICC=0.928) when measuring AHi (Figures 1e, 1f). Among the traditional metrics reported by GS and RG, the AHi was thus the most reliable. This information is important since all three databases and the metrics they provide are freely available to registered users. However, Sc is the only curated database among the analyzed databases and is likely to be the most reliable.

The Google Scholar i-10-index

The author i10-index represents the number of papers authored by a researcher that have accrued at least 10 citations. We found that the i10-index had a strong positive correlation with the AHi, NP, and NC from Sc. We also found that the i10-index for a given author was always higher than the AHi (vide supra). This could be explained by the fact that whereas the AHi grows uniformly as NP and NC increase, the citation count component of the i10-index remains static at 10. Thus, the i10 could reach arbitrarily high values for prolific researchers who publish a larger number of papers (albeit poorly cited). There are two other situations where the i10 would be a suboptimal measure of author impact. First, the i10-index also does not compute for early-career clinicians whose research has not had enough time to accrue at least 10 citations. Second, if a researcher published a limited number of significant papers that then go on to be very highly cited, their i10-index would continue to remain relatively low. Thus, the i10-index is designed to “reward” prolific rather than impactful authors [16]. Hence, in all the mentioned scenarios, the AHi would be a better measure of research impact.

ResearchGate alternative metrics

The primary alternative metrics offered by ResearchGate are the RG score and Research Interest. The stated variables used to compute the RG score are NP, questions, answers, and followers. The RG score displayed a robust correlation with the traditional metrics; this has been previously reported for other disciplines as well [17]. However, when we performed curve estimation analyses, we found that the RG score displayed a logarithmic relation with Sc-NP, Sc-NC, and Sc-AHi, approaching an asymptote beyond an RG score of 50 (Figures 2a-2c). This implies that the RG score reaches a plateau at approximately 50 and could be unreliable for the more senior authors. On the other hand, at low values of the RG score, even a small change in AHi or NP results in a significant change in the RG score. Thus, for both early-career researchers (lower NP, NC, and AHi) and the titans (high NP, NC, and AHi), the RG score may not be an accurate and fair measure of scholarly impact. It has been previously reported that one way to significantly boost the RG score beyond 50 is by answering questions on RG [18,19]. In the present cohort of JNS authors, however, fewer than 100 had answered a question on their profile. Thus, answering questions on RG is unlikely to be a significant factor in determining the RG score of neurosurgeons.

Research Interest is apparently computed using four components: a read, with a weighting of 0.05, a full text read with a weighting of 0.15, a recommendation with a weighting of 0.25, and a citation with a weighting of 0.5. It was not possible for us to replicate the computation of the Research Interest metric since the number of full text reads for authors is not publicly available. There was a strong positive correlation between Sc-NC and Research Interest (⍴=0.931) as well as between RG-NC and Research Interest (⍴=0.993). The extremely high degree of correlation between citations and Research Interest could imply that NC has a significant weightage in its computation [5].

RG reads is another metric provided by ResearchGate. A "read" is counted each time a user views the abstract, clicks on a figure linked to the publication, or views or downloads the full text of a paper. In the present analysis, we found a strong positive correlation between RG reads and the three traditional metrics (Table 3). Since the authors themselves receive a personalized breakdown of reader demographics for individual research items, RG reads is a good platform engagement metric. 

"Followers" is the number of RG users who follow a specific researcher. We found that the number of RG followers correlated with Sc-NP, NC, and AHi (Table 3). Authors with high impact in their respective fields also tend to have higher values of traditional metrics and thus users on RG tend to follow such authors when present. Therefore, a researcher’s follower count could effectively serve as a surrogate measure of research reputation. 

The author Impact Matrix

We found that RG score, Research Interest, and RG followers were the novel metrics that independently predicted Sc-NC and Sc-AHi in the multivariate analyses. We would thus suggest using AHi as the traditional metric of choice where applicable. The RG score, Research Interest score, and the number of RG followers taken together could add dimensions that describe the real-world impact of an author’s research. 

**Figure 3:**
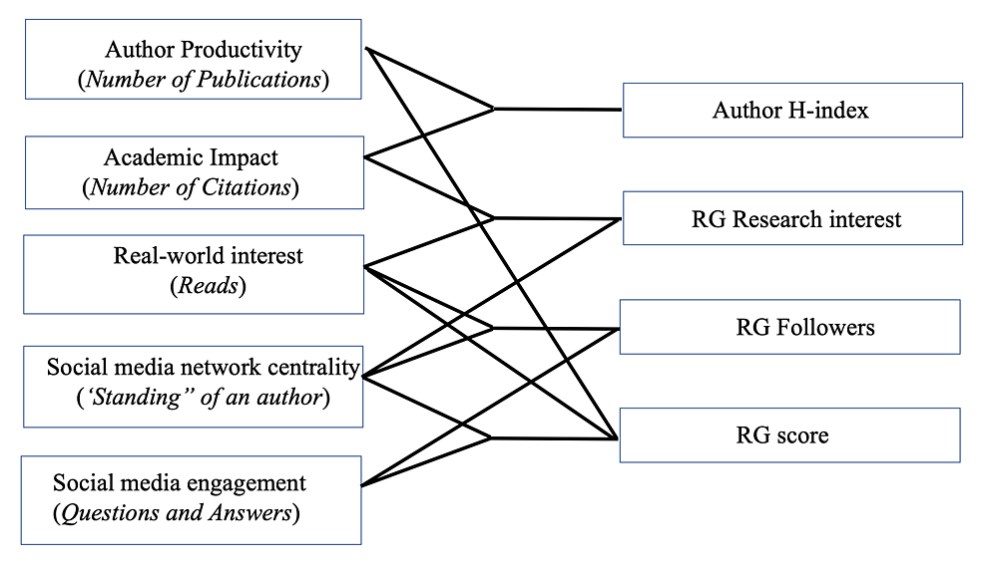
The four-dimensional author Impact Matrix The boxes on the left depict the various axes that determine an author’s impact and the boxes on the right depict the metrics that form the author Impact Matrix

These four metrics represent various axes that together determine the entirety of the impact of an author’s work. A composite author-level matrix including these four metrics could be used to effectively describe the scholarly and real-world impact of a clinician (Figure 3).

The four "dimensions," their constituent axes, and their implications are as follows:

Dimension 1: AHi 

The AHi combines two measures (axes): (a) research/publication productivity of an author, measured by the NP, and (b) the impact of an author’s work on other academic writers/researchers, measured by the NC. Thus, the AHi describes both the quantity and quality of an author’s research, with a special focus on its utility to other researchers. This is the simplest dimension, incorporating only two axes. 

Dimension 2: Research Interest Score

This metric combines three axes: (a) academic impact - NC, (b) real-world impact - reflected by the number of reads and unmeasurable changes in practice, and (c) social media standing of an author, reflected by the number of recommendations, etc.

Dimension 3: Number of Followers

This metric also incorporates three axes: (a) real-world impact, (b) social media standing of an author, and (c) engagement with social media - reflected by the number of questions and answers, etc. 

Dimension 4: RG Score

This dimension is represented by a composite impact metric. The RG score incorporates the following axes: (a) the productivity of an author (NP), the number of followers, which itself incorporates (b) real-world impact, (c) social media standing of an author, and (d) engagement with social media - reflected by the number of questions and answers, etc. Thus, this is the most complex dimension that incorporates four axes.

Thus, these dimensions could be considered to form a four-dimensional author “Impact Matrix” for every individual author/researcher (Figure 3). 

In the future, it is possible that a composite matrix akin to the one we have described here would be used to gauge the contributions of a clinician. Such an Impact Matrix would more efficiently gauge the impact of a clinician or researcher across academia as well as the real world. However, it must be said that even this matrix fails to capture the clinical productivity and impact of a physician - and this would be of particular importance in a surgical field such as neurosurgery.

Global perspective

In our analysis of the traditional author-level metrics from different continents, we found that authors from Asia (despite constituting only 20% of the cohort of JNS authors for 2019) had the highest median number of publications. This is likely due to the fact that Asian countries such as China, India, Japan, and South Korea have gradually been improving their clinical and research output. A similar skew in the volume of research output has been seen in other specialties as well [20]. However, the highest median AHi was seen for European authors, indicating that the European authors who had published in 2019 in JNS were the most impactful. 

The highest median values of the alternative metrics that purport to measure reputation, such as the RG score and RG Research Interest, were seen for European authors. This is likely due to the fact that European authors also had the highest median values of the traditional metrics and the calculation of the alternative metrics is dependent upon traditional metrics to some degree. On the contrary, South American neurosurgeons/authors had the highest median values of those alternative metrics that measured platform engagement (such as RG reads, recommendations, followers, and following). This could imply that South American neurosurgeons/authors utilized ResearchGate more as a networking platform than did neurosurgeons from other continents [21]. Fundamental differences in publishing practices, scholarly impact, and the usage of social media research platforms appear to exist among neurosurgeons from different continents. A higher degree of collaboration among authors from different countries, facilitated by social media, which could be used both for networking as well as for active knowledge dissemination, would lead to improvements in the quality and quantity of published neurosurgical research [22,23].

Limitations of the study

This study only included authors who published in JNS. Although JNS is one of the most prominent journals that publish neurosurgical literature, the inclusion of other high-impact neurosurgical and clinical neuroscience journals in the analysis would have greatly improved the generalizability of our findings. Secondly, owing to the size of the dataset, the present study was designed to only include authors who had published in the year 2019. Thus, we were unable to carry out a longitudinal citation trends analysis.

Comparing NP across databases is purely dependent on how many journals are represented in the said database. However, JNS*, *which was the only journal included in this analysis, was included in all the databases analyzed in this study.

Finally, working with publicly available databases proved to be a challenge since all the necessary data points were not available for all the authors. For instance, we had a smaller sample of GS metrics due to IP address restrictions. The use of Harzing's Publish or Perish software as an alternative returned several duplicate entries and filtering based on author names was not entirely effective. These limitations limited the sample size and the data points available.

Another point to be noted is the fact that none of the traditional or alternative metrics or comparisons can gauge the relative contribution of an author in a publication. However, considering that the same authors who published in JNS would also be likely to publish in the other high-impact neurosurgical journals, we believe that the results of the present study are robust and valid.

## Conclusions

This study is the first comprehensive analysis of the traditional and alternative metrics used to measure scholarly impact, applied to neurosurgeons and authors from related disciplines, who had published in JNS. The AHi was the most consistent and reliable metric across databases. Considered in conjunction with the AHi, the alternative ResearchGate metrics (RG score, Research Interest, and the number of followers) can be used to create an author Impact Matrix that could provide a comprehensive picture of an author’s academic and real-world impact.

## References

[1] Harzing AW, Alakangas S (2016). Google Scholar, Scopus and the Web of Science: a longitudinal and cross-disciplinary comparison. Scientometrics.

[2] Bakkalbasi N, Bauer K, Glover J, Wang L (2006). Three options for citation tracking: Google Scholar, Scopus and Web of Science. Biomed Digit Libr.

[3] Koltun V, Hafner D (2021). The h-index is no longer an effective correlate of scientific reputation. PLoS One.

[4] Kraker Kraker, P. P., & Lex, E E (2022). Graz University of Technology: a critical look at the ResearchGate score as a measure of scientific reputation. Proceedings of.

[5] Copiello S (2019). Research Interest: another undisclosed (and redundant) algorithm by ResearchGate. Scientometrics.

[6] Jamali HR, Nicholas D, Herman E (2015). Scholarly reputation in the digital age and the role of emerging platforms and mechanisms. Res Eval.

[7] Ban VS, Lega B, Batjer HH (2016). Maximizing the potential of social media and social networks in neurosurgery. World Neurosurg.

[8] Alotaibi NM, Guha D, Fallah A (2016). Social media metrics and bibliometric profiles of neurosurgical departments and journals: is there a relationship?. World Neurosurg.

[9] Bar-Ilan J (2008). Which h-index?—a comparison of WoS, Scopus and Google Scholar. Scientometrics.

[10] Walker B, Alavifard S, Roberts S, Lanes A, Ramsay T, Boet S (2016). Inter-rater reliability of h-index scores calculated by Web of Science and Scopus for clinical epidemiology scientists. Health Info Libr J.

[11] (2022). Scopus Coverage Content Guide. https://www.elsevier.com/?a=69451.

[12] Burghardt KJ, Howlett BH, Khoury AS, Fern SM, Burghardt PR (2020). Three commonly utilized scholarly databases and a social network site provide different, but related, metrics of pharmacy faculty publication. Publications.

[13] Alcaraz C, Morais S (2012). Citations: results differ by database. Nature.

[14] López-Cózar ED, Robinson-García N, Torres-Salinas D (2012). Manipulating Google Scholar citations and Google Scholar metrics: simple, easy and tempting. EC3 Working Papers.

[15] Beel J, Gipp B (2010). Academic search engine spam and Google Scholar’s resilience against it. J Electron Publ.

[16] da Silva JAT (2021). The i100-index, i1000-index and i10, 000-index: expansion and fortification of the Google Scholar h-index for finer-scale citation descriptions and researcher classification. Scientometrics.

[17] Joshi ND, Lieber B, Wong K, Al-Alam E, Agarwal N, Diaz V (2019). Social media in neurosurgery: using ResearchGate. World Neurosurg.

[18] Nicholas D, Clark D, Herman E (2016). ResearchGate: reputation uncovered. Learn Publ.

[19] Orduna-Malea E, Martín-Martín A, Thelwall M, López-Cózar ED (2017). Do ResearchGate Scores create ghost academic reputations?. Scientometrics.

[20] Hu X, Rousseau R (2009). A comparative study of the difference in research performance in biomedical fields among selected Western and Asian countries. Scientometrics.

[21] Thelwall M, Kousha K (2015). ResearchGate: disseminating, communicating, and measuring scholarship?. J Assoc Inf Sci Technol.

[22] Niquen-Jimenez M, Wishart D, Garcia RM (2020). A bibliographic analysis of the most cited articles in global neurosurgery. World Neurosurg.

[23] Wang J, Alotaibi NM, Ibrahim GM, Kulkarni AV, Lozano AM (2017). The spectrum of Altmetrics in neurosurgery: the top 100 "trending" articles in neurosurgical journals. World Neurosurg.

